# Impacts of double biopsy and double vitrification on the clinical outcomes following euploid blastocyst transfer: a systematic review and meta-analysis

**DOI:** 10.1093/humrep/deae235

**Published:** 2024-10-07

**Authors:** Kate Bickendorf, Fang Qi, Kelli Peirce, Rui Wang, Jay Natalwala, Vincent Chapple, Yanhe Liu

**Affiliations:** Fertility North, Joondalup Private Hospital, Joondalup, WA, Australia; Research & Development Department, Systematic Review Solutions Ltd, Shanghai, China; Fertility North, Joondalup Private Hospital, Joondalup, WA, Australia; NHMRC Clinical Trials Centre, University of Sydney, Sydney, NSW, Australia; Department of Obstetrics and Gynaecology, Monash University, Clayton, Australia; Fertility North, Joondalup Private Hospital, Joondalup, WA, Australia; Fertility North, Joondalup Private Hospital, Joondalup, WA, Australia; Fertility North, Joondalup Private Hospital, Joondalup, WA, Australia; School of Medical and Health Sciences, Edith Cowan University, Joondalup, WA, Australia; School of Human Sciences, University of Western Australia, Crawley, WA, Australia

**Keywords:** blastocyst, biopsy, trophectoderm, meta-analysis, preimplantation genetic testing, vitrification, test failure, pregnancy, birth, survival rate

## Abstract

**STUDY QUESTION:**

Compared to the ‘single biopsy + single vitrification’ approach, do ‘double biopsy + double vitrification’ or ‘single biopsy + double vitrification’ arrangements compromise subsequent clinical outcomes following euploidy blastocyst transfer?

**SUMMARY ANSWER:**

Both ‘double biopsy + double vitrification’ and ‘single biopsy + double vitrification’ led to reduced live birth/ongoing pregnancy rates and clinical pregnancy rates.

**WHAT IS KNOWN ALREADY?:**

It is not uncommon to receive inconclusive results following blastocyst biopsy and preimplantation genetic testing for aneuploidy (PGT-A). Often these blastocysts are warmed for re-test after a second biopsy, experiencing ‘double biopsy + double vitrification’. Furthermore, to achieve better workflow, IVF laboratories may choose to routinely vitrify all blastocysts and schedule biopsy at a preferred timing, involving ‘single biopsy + double vitrification’. However, in the current literature, there is a lack of systematic evaluation of both arrangements regarding their potential clinical risks in reference to the most common ‘single biopsy + single vitrification’ approach.

**STUDY DESIGN, SIZE, DURATION:**

A systematic review and meta-analysis were performed, with the protocol registered in PROSPERO (CRD42023469143). A search in PUBMED, EMBASE, and the Cochrane Library for relevant studies was carried out on 30 August 2023, using the keywords ‘biopsy’ and ‘vitrification’ and associated variations respectively. Only studies involving frozen transfers of PGT-A tested euploid blastocysts were included, with those involving PGT-M or PGT-SR excluded.

**PARTICIPANTS/MATERIALS, SETTING, METHODS:**

Study groups included blastocysts having undergone ‘double biopsy + double vitrification’ or ‘single biopsy + double vitrification’, with a ‘single biopsy + single vitrification’ group used as control. The primary outcome was clinical pregnancy, while secondary outcomes included live birth/ongoing pregnancy, miscarriage, and post-warming survival rates. Random effects meta-analysis was performed with risk ratios (RR) and 95% CIs were used to present outcome comparisons.

**MAIN RESULTS AND THE ROLE OF CHANCE:**

A total of 607 records were identified through the initial search and nine studies (six full articles and three abstracts) were eventually included. Compared to ‘single biopsy + single vitrification’, ‘double biopsy + double vitrification’ was associated with reduced clinical pregnancy rates (six studies, n = 18 754; RR = 0.80, 95% CI = 0.71–0.89; *I*^2^ = 0%) and live birth/ongoing pregnancy rates (seven studies, n = 20 964; RR = 0.72, 95% CI = 0.63–0.82; *I*^2^ = 0%). However, no significant changes were seen in miscarriage rates (seven studies, n = 22 332; RR = 1.40, 95% CI = 0.92–2.11; *I*^2^ = 53%) and post-warming survival rates (three studies, n = 13 562; RR = 1.00, 95% CI = 0.99–1.01; *I*^2^ = 0%) following ‘double biopsy + double vitrification’. Furthermore, ‘single biopsy + double vitrification’ was also linked with decreased clinical pregnancy rates (six studies, n = 13 284; RR = 0.84, 95% CI = 0.76–0.92; *I*^2^ = 39%) and live birth/ongoing pregnancy rates (seven studies, n = 16 800; RR = 0.79, 95% CI = 0.69–0.91; *I*^2^ = 70%), and increased miscarriage rates (five studies, n = 15 781; RR = 1.48, 95% CI = 1.31–1.67; *I*^2^ = 0%), but post-warming survival rates were not affected (three studies, n = 12 452; RR = 0.99, 95% CI = 0.97–1.01; *I*^2^ = 71%) by ‘single biopsy + double vitrification’.

**LIMITATIONS, REASONS FOR CAUTION:**

All studies included in this meta-analysis were retrospective with varying levels of heterogeneity for different outcomes. Not all studies had accounted for potential confounding factors. Only one study reported neonatal outcomes.

**WIDER IMPLICATIONS OF THE FINDINGS:**

Our data indicated adverse impacts of ‘double biopsy + double vitrification’ and ‘single biopsy + double vitrification’ on clinical outcomes following euploid blastocyst transfers. Patients should be carefully consulted about the risks when offered such approaches. The biopsy process should be carried out as carefully and competently as possible to minimize an inconclusive diagnosis.

**STUDY FUNDING/COMPETING INTEREST(S):**

R.W. is supported by a National Health and Medical Research Council Emerging Leadership Investigator Grant (2009767). There is no other external funding to report. All authors report no conflict of interest.

**REGISTRATION NUMBER:**

CRD42023469143.

## Introduction

In IVF laboratories, blastocyst biopsy is becoming a standard procedure where indicated, despite the involvement of invasive removal of several trophectoderm cells for genetic testing (Nohales *et al.*, 2023). Pioneered by Handyside *et al.* (1990), preimplantation genetic testing for aneuploidy (PGT-A) has been increasingly used to improve the success rate per embryo transfer, whereby only embryos testing as euploid are identified and selected for transfer (Sciorio *et al.*, 2020). The common occurrence of abnormal chromosome numbers in the embryo, also known as aneuploidy, is a major factor in failed implantation and miscarriage (Kimelman and Pavone, 2021).

While PGT-A testing is complementary to IVF, it is not unusual that a conclusive diagnosis fails to be achieved. Different studies have reported various rates of inconclusive diagnosis ranging from 0.86% to 6.3% (Fiorentino *et al.*, 2014; Capalbo *et al.*, 2016; Cimadomo *et al.*, 2018; Neal *et al.*, 2019). In a comprehensive investigation performed by Cimadomo *et al.* (2018), failures in obtaining conclusive diagnosis were found to be caused by factors including technical amplification failure of DNA, failure to meet analytical quality metrics due to a wide scatter in the data, poor quality DNA that is secondary to poor embryo quality, the day of biopsy (Days 5–7), biopsy technique/method (e.g. flicking or pulling), and shipping conditions. Nohales *et al.* (2023) expanded on this, highlighting that extra cell damage can be related to laboratory practice, poor biopsy technique, and the process of sample handling. An optimal balance must be maintained, where not only trauma introduced by biopsy to the blastocyst is minimized, but also the integrity of the sampled trophectoderm cells is maximized. Apart from transferring these blastocysts without a conclusive diagnosis, an alternative approach is to re-biopsy for a re-test, with the involvement of an additional round of vitrification and warming of the blastocysts. As such, even if the blastocyst eventually tests as euploid, further compromise in its viability could have already occurred, considering the loss of additional trophectoderm cells via double-biopsy and extracellular stress induced by the repeated temperature change during double-vitrification. However, there is currently a lack of consensus regarding the level of impacts, if any, of such ‘double biopsy + double vitrification’ on the subsequent clinical outcomes following transfers of blastocysts tested for euploidy.

The global move from slow freezing to vitrification of blastocysts has boosted the post-thaw/warming survival rate to as high as 95–99% (Rienzi *et al.*, 2017). Supported by such success, double vitrification allows access to PGT-A by patients who have embryos already in storage but have not previously considered PGT-A (Wilding *et al.*, 2019). This ‘single biopsy + double vitrification’ approach is also thought to be workflow-friendly, especially in clinics with only a limited number of biopsy operators. By routinely offering such an arrangement to all PGT-A patients, biopsy procedures may be scheduled on the best-staffed days, avoiding weekends or public holidays. However, the combined impacts of two vitrification steps plus a biopsy procedure in between are still poorly understood. In contrast with this approach, the ‘double biopsy + double vitrification’ approach is mostly used in cases of failed PGT-A diagnosis, and considered only in specific cases where a final diagnosis has been requested or required.

Existing studies on this topic have shown conflicting conclusions (Wilding *et al.*, 2019; Makhijani *et al.*, 2021; Brolinson *et al.*, 2023; Nohales *et al.*, 2023). A systematic review of clinical outcomes following transfers of euploid blastocysts after either ‘double biopsy + double vitrification’ or ‘single biopsy + double vitrification’ is needed to assist with interpreting the conflicting findings and to identify the clinical and research gaps. This systematic review and meta-analysis aims to comprehensively evaluate clinical outcomes of blastocysts involved in the above-mentioned two arrangements, by comparisons to ‘single biopsy + single vitrification’.

## Materials and methods

The protocol of this systematic review and meta-analysis was registered with PROSPERO (CRD42023469143) (Bickendorf *et al.*, 2023). The review was reported in accordance with the Preferred Reporting Items for Systematic Reviews and Meta-Analyses (PRISMA) criteria (Page *et al.*, 2021).

### Search strategies

We searched PubMed, Embase, and the Cochrane Library on 30 August 2023 using the keywords ‘biopsy’ (‘trophectoderm biopsy’ or ‘re-biopsy’ or ‘double biopsy’ or ‘repeat biopsy’) and ‘vitrification’ (‘cryopreservation’ or ‘freeze’), with no restrictions on date, language, document type, or publication status. The detailed search strategy for each database is presented in the Supplementary Data File S1.

### Eligibility criteria and study selection

All prospective or retrospective observational comparative studies fulfilling the following criteria were included: (1) women undergoing frozen embryo transfers of euploid blastocyst(s) tested via PGT-A, but not preimplantation genetic testing for monogenetic disease (PGT-M) or structural rearrangement (PGT-SR), to avoid confounding effect by patient populations with different genetic/chromosomal profiles; (2) blastocysts biopsied 5/6/7 days post-oocyte collection followed by vitrification; and (3) a comparator group available where blastocysts were biopsied and vitrified once only. No limitation was applied to female age, sperm source (own/donor), and day of assisted hatching.

Comparisons were made for ‘double biopsy + double vitrification’ versus ‘single biopsy + single vitrification’ and for ‘single biopsy + double vitrification’ versus ‘single biopsy + single vitrification’, respectively. The primary outcome was clinical pregnancy and secondary outcomes were live birth or ongoing pregnancy, miscarriage, post-warming survival, and neonatal outcomes (including gestational age and birthweight).

The search results were independently screened by two reviewers (K.B. and F.Q.). After removing duplicate records and initially screening the remaining references by title and abstract, full-text reports of the references that appeared to meet the eligibility criteria were obtained and further reviewed for a final decision for inclusion. Any disagreement was resolved through discussion between the two reviewers, with assistance from a third reviewer (Y.L.) as necessary.

### Data extraction and quality assessment

Quantitative and qualitative information on participants, comparisons, and outcomes were extracted independently by two reviewers (K.B. and F.Q.) using a standardized data collection Excel form (Microsoft^®^ Excel^®^, Microsoft Corporation, Redmond, WA, USA). The Quality In Prognosis Studies tool (Hayden *et al.*, 2013) was used to assess the quality of included studies, including study participation, study attrition, prognostic factor measurement, outcome measurement, study confounding, statistical analysis, and reporting.

### Data analysis

For dichotomous outcomes, we presented the effect as a summary risk ratio (RR) or odds ratio (OR) with 95% CI. For continuous outcomes, we presented the effect as a summary mean difference (MD) with 95% CI. For the adjusted analysis, we planned to pool the adjusted RR or ORs as long as the key prognostic factors were adjusted. We also pooled the unadjusted RRs or ORs. Meta-analyses were conducted using RevMan 5.4 software, and the random effects model was used. *I*^2^ was used to present the percentage of total variability due to between-study heterogeneity. To investigate the sources of the heterogeneity, we planned to perform subgroup analyses based on female age, day of initial biopsy, or biopsy technique. However, these analyses were not performed as planned due to insufficient reported data. Contour-enhanced funnel plots were presented to demonstrate small study effects for the primary outcome.

## Results

### Study selection

**Figure 1.**
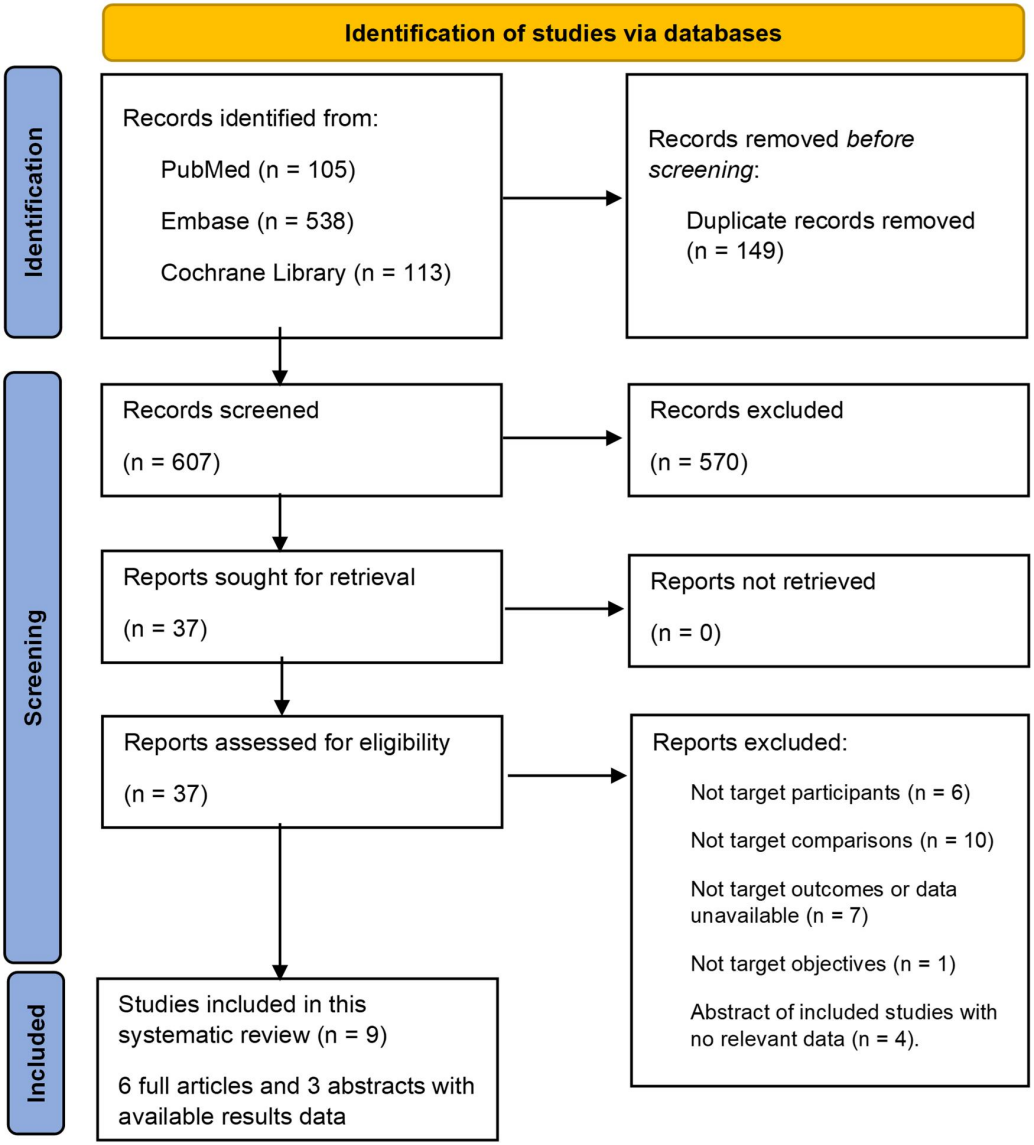
**PRISMA flow diagram of the identification of studies**. PRISMA, Preferred Reporting Items for Systematic Reviews and Meta-Analyses.

The search initially yielded 756 records across all databases. After removing duplicates, 607 unique records remained for screening. Upon inspection of titles and abstracts, 570 records were excluded. The remaining 37 records were read in full, with 28 subsequently excluded for various reasons as specified in Fig. 1 and Supplementary Data File S1. Finally, nine studies, consisting of six with full text (Cimadomo *et al.*, 2018; Neal *et al.*, 2019; Wilding *et al.*, 2019; Aluko *et al.*, 2021; Nohales *et al.*, 2023; Vanderhoff *et al.*, 2024) and three with abstract only (Gunnala *et al.*, 2018; Schlenker *et al.*, 2019; Brolinson *et al.*, 2023), were included in this systematic review and meta-analysis.

### Study characteristics and quality assessment

The characteristics of the included studies (reporting a total of 24 158 cycles) are presented in Table 1. A total of 407 transfers were included in the ‘double biopsy + double vitrification’ group, 776 transfers were included in ‘single biopsy + double vitrification’ group, and 22 975 transfers were included in ‘single biopsy + single vitrification’ group. All included studies were retrospective cohort studies with a moderate risk of bias overall (Supplementary Table S1). As the included studies were all retrospective in design, the ‘study participation’ and ‘study attrition’ domains were rated as moderate risk. Four studies considered key confounders and adjusted them in the analyses. They were rated at a low risk of bias for confounding and statistical analysis but were rated at a moderate risk of bias for unadjusted analysis.

**Table 1.** Characteristics of included studies.

Study ID	Groups included			Maternal age at oocyte collection (years, mean±SD, range)	Maternal age at embryo transfer (years, mean ±SD, range)	Maternal BMI (kg/m^2^, mean±SD, range)	Parity	Gravidity	Endometrial thickness at embryo transfer (mm, mean±SD, range)
	Double biopsy and double vitrification (n)	Single biopsy and double vitrification (n)	Single biopsy and single vitrification (n)						
Aluko2021F	15	95	2603	37.0 (34.9∼38.8); 33.9 (31.7∼37.7); 36.2 (33.5∼38.9)	37.5 (35.1∼39.1); 35.0 (32.0∼39.1); 36.7 (33.8∼39.3)	24.9 (23.1∼27.5); 24.0 (21.6∼27.5); 24.0 (21.6∼27.5)	0: 8 (53.3%); 67 (70.5%); 2202 (84.6%) 1: 7 (46.7%); 24 (25.3%); 308 (11.8%) 2+: 0 (0%); 4 (4.2%); 93 (3.6%)	0: 8 (53.3%); 40 (42.1%); 1865 (71.6%) 1: 2 (13.3%); 21 (22.1%); 263 (10.1%) 2+: 5 (33.3%); 34 (35.8%); 475 (18.2%)	8.5 (7.7∼12.1); 8.8 (7.9∼10.2); 8.5 (7.7∼12.1)
Brolinson2023A	124	599^a^	7238	NR	NR	NR	NR	NR	NR
Cimadomo2018F	49	×	2825	NR	NR	NR	NR	NR	NR
Gunnala2018A	×	125	1028	34.4 ± 4.2; 36.3 ± 4.1	NR	24.0 ± 6.5; 23.4 ± 4.3	Median: 1; 0	Median: 3; 2	9.4 ± 1.8; 9.1 ± 1.8
Neal2019F	36	155	3542	35.5 ± 4.4; 32.0 ± 4.4; 34.8 ± 4.4	36.2 ± 4.4; 35.8 ± 5.6; 36.0 ± 4.4	26.0 ± 5.8; 26.5 ± 5.6; 26.1 ± 5.6	NR	NR	11.0 ± 2.4; 10.6 ± 2.8; 10.5 ± 2.5
Nohales2023F	71	×	4562	<35: 81; 3069 35–37: 49; 1491 38–40: 69; 2278 41–42: 21; 630 >42: 4; 135	NR	NR	NR	NR	NR
Schlenker2019A	93	326	93	NR	37.8 ± 3.8; 35.1 ± 3.7; 37.3 ± 3.4	NR	NR	NR	NR
Vanderhoff2024F	19	68	1062	37.0 ± 4.02; 35.64 ± 3.59; 37.12 ± 4.00	NR	24.63 ± 6.81; 24.62 ± 5.10; 25.06 ± 5.75	NR	NR	NR
Wilding2019F	×	21	26	NR	38.1 ± 4.1; 40.1 ± 3.6	NR	NR	NR	NR

Study ID	Day of freeze (5/6/7)	Day 3 assisted hatching	Prevalence of undiagnosed embryo	Number of blastocysts transferred (mean±SD)	Outcome reported	Definition of outcome	Included in the meta-analysis (Y/N)	Results data presented
Aluko2021F	Day 5: 11 (73.3%); 80 (84.2%); 1528 (58.7%) Day 6: 4 (26.7%); 15 (15.8%); 1058 (40.6%) Day 7: 0 (0%); 0 (0%); 17 (0.7%)	NP	2.3% (386/16 789)	1	Clinical pregnancy rate	a sonographic intrauterine pregnancy with a gestational sac	Y	Fig. 2; Supplementary Fig. S2
					Live birth rate	NR	Y	Fig. 3; Supplementary Fig. S2
					Miscarriage rate	NR	Y	Fig. 4; Supplementary Fig. S2
					Post-warm survival rate	NR	Y	Fig. 5
Brolinson2023A	NR	NP	NP	NR	Clinical pregnancy rate	NR	Y	Fig. 2
					Live birth and ongoing pregnancy rate	NR	Y	Fig. 3
					Miscarriage rate	NR	Y (Categorized as ‘Miscarriage rate’)	Fig. 4
					Post-warm survival rate	NR	Y	Fig. 5
Cimadomo2018F	NR	N (hatch right before biopsy)	2.5% (228/8990)	1	Clinical pregnancy rate	gestational sacs with foetal heartbeat/transferred blastocysts	Y	Fig. 2
					Live birth rate	live births/transferred blastocysts	Y	Fig. 3
					Miscarriage rate	Miscarriages/transferred blastocysts	Y	Fig. 4
					Post-warm survival rate	NR	Y	Fig. 5
					Gestational age	NR	N	Table 2
					Birthweight	NR	N	Table 2
Gunnala2018A	NR	NR	NR	1.1 ± 0.4; 1.2 ± 0.4	Ongoing pregnancy rate	NR	Y	Fig. 3
					Clinical pregnancy rate	NR	Y	Fig. 2
					Post-warm survival rate	NR	Y	Fig. 5
Neal2019F	Day 5: 13 (36.1%); 63 (40.6%); 1765 (49.8%) Day 6: 22 (61.1%); 91 (58.7%); 1723 (48.7%) Day 7: 1 (2.8%); 1 (0.6%); 54 (1.5%)	NR	2.5% (635/25 199)	1	Ongoing pregnancy rate	the presence of a foetal heartbeat at 8-week gestation	Y	Fig. 3
					Clinical loss rate	pregnancy loss following observation of a gestational sac on ultrasound	Y (Categorized as ‘Miscarriage rate’)	Fig. 4; Supplementary Fig. S2
Nohales2023F	NR	Y	2.9% (517/18 028)	1	Clinical pregnancy rate	the percentage of embryo transfers resulting in a gestational sac observed during the ultrasound examination scan at >5 weeks gestation	Y	Fig. 2
					Live birth rate	the percentage of embryo transfers resulting in a foetus born alive beyond the 24th week of pregnancy	Y	Fig. 3
					Miscarriage rate	the number of miscarriages up to the 20th week of pregnancy divided by the number of gestations with positive β-hCG	Y	Fig. 4
Schlenker2019A	NR	NR	NR	NR	Clinical pregnancy rate	with foetal cardiac activity	Y	Fig. 2
					Live birth rate	NR	Y	Fig. 3
					Miscarriage rate	NR	Y	Fig. 4
Vanderhoff2024F	Day 5: 15 (78.95%); 62 (91.18%); 1033 (94.94%) Day 6: 4 (21.05%); 6 (8.82%); 54 (4.96%) Day 7: 0 (0%); 0 (0%); 1 (0.09%)	Y	2.46% (169/6858)	1	Ongoing pregnancy rate	NR	Y	Fig. 3; Supplementary Fig. S2
					Live birth rate	NR	Y	Fig. 3; Supplementary Fig. S2
					Miscarriage rate	NR	Y	Fig. 4
Wilding2019F	NR	NR	4.7% (20/427)	1.05 ± 0.4; 1.0 ± 0.0	Clinical pregnancy rate	NR	Y	Fig. 2
					Live birth rate	NR	Y	Fig. 3

a StudyID, A presents for abstract only, F presents for full-text available; n, number of cycles; Y, yes; N, no; NR, not reported. To align across studies, number of cycles was used as denominator instead of number of clinical pregnancies, when necessary, in the following studies: Cimadomo2018F, Neal2019F, Nohales2023F, Wilding2019F. The number of cycles in ‘single biopsy and double vitrification’ group was calculated by the sum number of cycle outcomes in Brolinson2023A (Table 1, 599 = 310 + 95 + 2+192). Gunnala2018A and Nohales2023F did not report age at oocyte collection or embryo transfer; The ‘number of blastocysts transferred’ equals one for ‘single blastocyst transferred’.

### Meta-analyses for all outcomes

#### Clinical pregnancy

**Figure 2.**
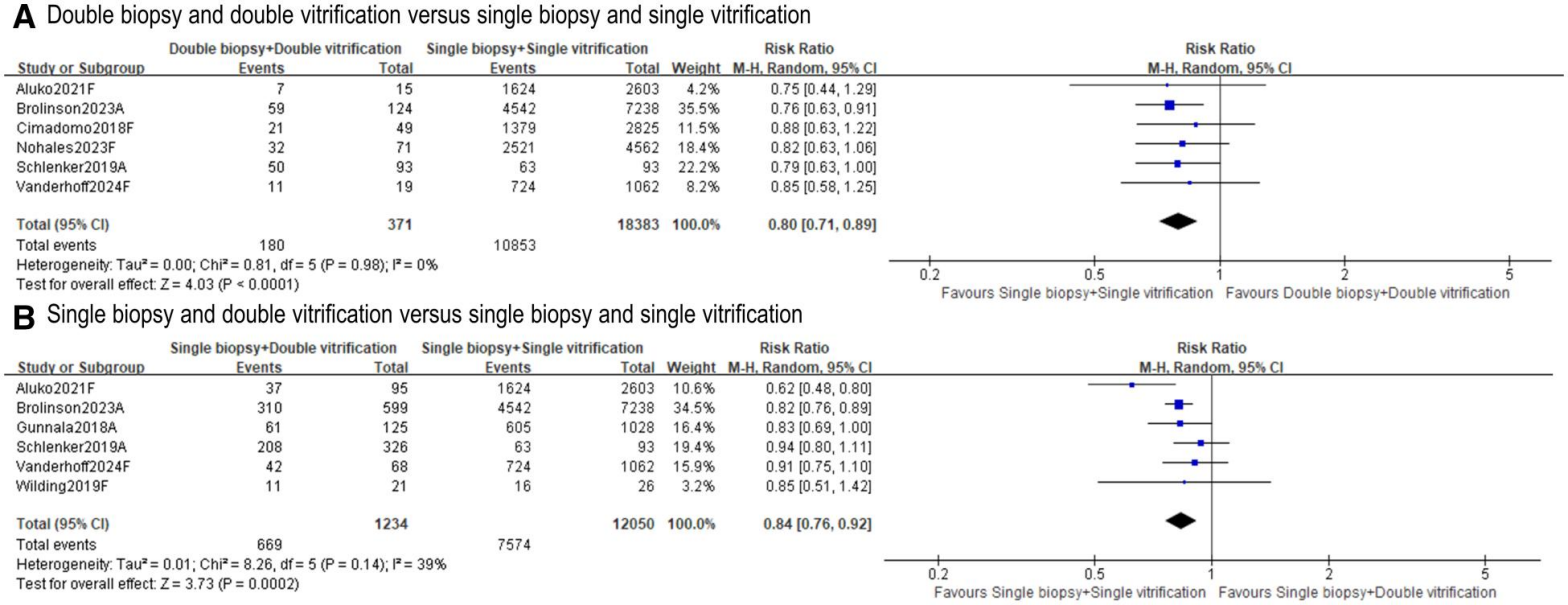
**Effects of ‘double biopsy+double vitrification’ and ‘single biopsy+double vitrification’ on clinical pregnancy rates**. **A.** Clinical pregnancy rate following ‘double biopsy and double vitrification’ versus ‘single biopsy and single vitrification’; **B.** Clinical pregnancy rate following ‘single biopsy and double vitrification’ versus ‘single biopsy and single vitrification’. Study ID: A presents for abstract only, F presents for full-text available. ‘Total’ represents the number of cycles.

Eight included studies (Cimadomo *et al.*, 2018; Gunnala *et al.*, 2018; Schlenker *et al.*, 2019; Wilding *et al.*, 2019; Aluko *et al.*, 2021; Brolinson *et al.*, 2023; Nohales *et al.*, 2023; Vanderhoff *et al.*, 2024) reported clinical pregnancy rates. The ‘double biopsy + double vitrification’ group resulted in lower clinical pregnancy rates than those in ‘single biopsy + single vitrification’ group (six studies, n = 18 754; RR 0.80, 95% CI = 0.71–0.89; *I*^2^ = 0%; Fig. 2A) (Cimadomo *et al.*, 2018; Schlenker *et al.*, 2019; Aluko *et al.*, 2021; Brolinson *et al.*, 2023; Nohales *et al.*, 2023; Vanderhoff *et al.*, 2024). A similar result was observed when comparing ‘single biopsy + double vitrification’ to ‘single biopsy + single vitrification’ (six studies, n = 13 284; RR 0.84, 95% CI = 0.76–0.92; *I*^2^ = 39%; Fig. 2B) (Gunnala *et al.*, 2018; Schlenker *et al.*, 2019; Wilding *et al.*, 2019; Aluko *et al.*, 2021; Brolinson *et al.*, 2023; Vanderhoff *et al.*, 2024). Contour-enhanced funnel plots (Supplementary Fig. S1) showed evidence of potential publication bias due to small study effects.

Only Aluko *et al.* (2021) reported results using a multivariable regression model, where a reduced clinical pregnancy rate was detected following ‘single biopsy + double vitrification’ but not following ‘double biopsy + double vitrification’ (Supplementary Fig. S2).

#### Live birth or ongoing pregnancy

**Figure 3.**
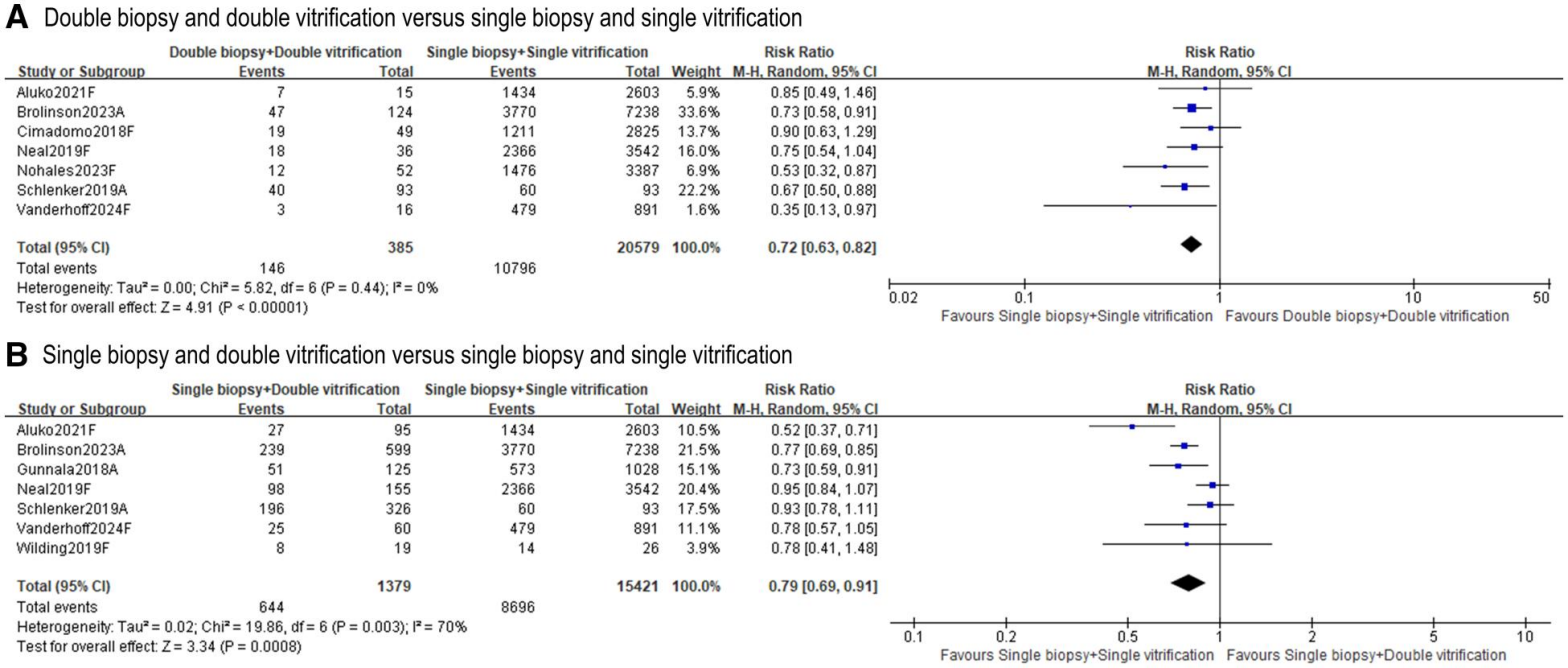
**Effects of ‘double biopsy+double vitrification’ and ‘single biopsy+double vitrification’ on live birth or ongoing pregnancy rates**. **A.** Live birth or ongoing pregnancy rate following ‘double biopsy and double vitrification’ versus ‘single biopsy and single vitrification’; **B.** Live birth or ongoing pregnancy rate following ‘single biopsy and double vitrification’ versus ‘single biopsy and single vitrification’. Study ID: A presents for abstract only, F presents for full-text available. ‘Total’ represents the number of cycles.

Six included studies reported live birth (Cimadomo *et al.*, 2018; Schlenker *et al.*, 2019; Wilding *et al.*, 2019; Aluko *et al.*, 2021; Nohales *et al.*, 2023; Vanderhoff *et al.*, 2024), three (Gunnala *et al.*, 2018; Neal *et al.*, 2019; Vanderhoff *et al.*, 2024) reported ongoing pregnancy rates, and one (Brolinson *et al.*, 2023) reported live birth or ongoing pregnancy rate as a composite outcome. Here, we report the pooled composite of outcome live birth or ongoing pregnancy rates. The ‘double biopsy + double vitrification’ group resulted in lower live birth/ongoing pregnancy rates than those in the ‘single biopsy + single vitrification’ group (seven studies, n = 20 964; RR 0.72, 95% CI = 0.63–0.82; *I*^2^ = 0%; Fig. 3A) (Cimadomo *et al.*, 2018; Neal *et al.*, 2019; Schlenker *et al.*, 2019; Aluko *et al.*, 2021; Brolinson *et al.*, 2023; Nohales *et al.*, 2023; Vanderhoff *et al.*, 2024). A similar trend was detected when comparing ‘single biopsy + double vitrification’ with ‘single biopsy + single vitrification’ (seven studies, n = 16 800; RR 0.79, 95% CI = 0.69–0.91; *I*^2^ = 70%; Fig. 3B) (Gunnala *et al.*, 2018; Neal *et al.*, 2019; Schlenker *et al.*, 2019; Wilding *et al.*, 2019; Aluko *et al.*, 2021; Brolinson *et al.*, 2023; Vanderhoff *et al.*, 2024). Three of the nine studies also analysed live birth/ongoing pregnancy rates using a multivariable regression model, and the pooled data indicated a reduced live birth or ongoing pregnancy rate following ‘double biopsy + double vitrification’ but not ’single biopsy + double vitrification’ (Supplementary Fig. S2) (Neal *et al.*, 2019; Aluko *et al.*, 2021; Vanderhoff *et al.*, 2024).

#### Miscarriage

**Figure 4.**
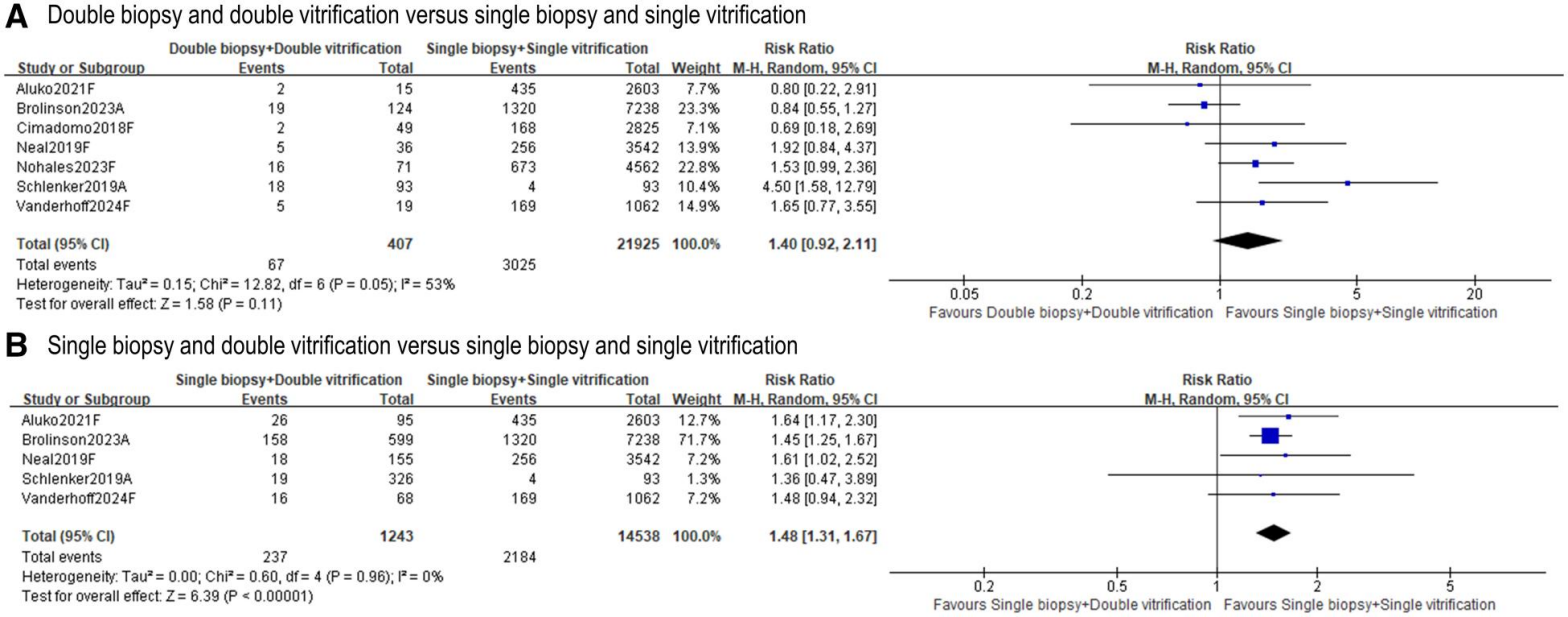
**Effects of ‘double biopsy+double vitrification’ and ‘single biopsy+double vitrification’ on miscarriage rates**. **A.** Miscarriage rate following ‘double biopsy and double vitrification’ versus ‘single biopsy and single vitrification’; **B.** Miscarriage rate following ‘single biopsy and double vitrification’ versus ‘single biopsy and single vitrification’. Study ID: A presents for abstract only, F presents for full-text available. ‘Total’ represents the number of cycles.

Seven included studies (Cimadomo *et al.*, 2018; Neal *et al.*, 2019; Schlenker *et al.*, 2019; Aluko *et al.*, 2021; Brolinson *et al.*, 2023; Nohales *et al.*, 2023; Vanderhoff *et al.*, 2024) reported miscarriage rates. The ‘double biopsy + double vitrification’ group resulted in similar miscarriage rates to the ‘single biopsy + single vitrification’ group (seven studies, n = 22 332; RR 1.40, 95% CI = 0.92–2.11; *I*^2^ = 53%; Fig. 4A) (Cimadomo *et al.*, 2018; Neal *et al.*, 2019; Schlenker *et al.*, 2019; Aluko *et al.*, 2021; Brolinson *et al.*, 2023; Nohales *et al.*, 2023; Vanderhoff *et al.*, 2024). However, elevated miscarriage rates were observed following ‘single biopsy + double vitrification’ in comparison to ‘single biopsy + single vitrification’ (five studies, n = 15 781; RR 1.48, 95% CI = 1.31–1.67; *I*^2^ = 0%; Fig. 4B) (Neal *et al.*, 2019; Schlenker *et al.*, 2019; Aluko *et al.*, 2021; Brolinson *et al.*, 2023; Vanderhoff *et al.*, 2024). Two of the seven studies evaluated miscarriage rates via multivariable regression, but no impact was identified following either ‘double biopsy + double vitrification’ or ’single biopsy + double vitrification’ (Supplementary Fig. S2) (Neal *et al.*, 2019; Aluko *et al.*, 2021).

#### Post-warming survival

**Figure 5.**
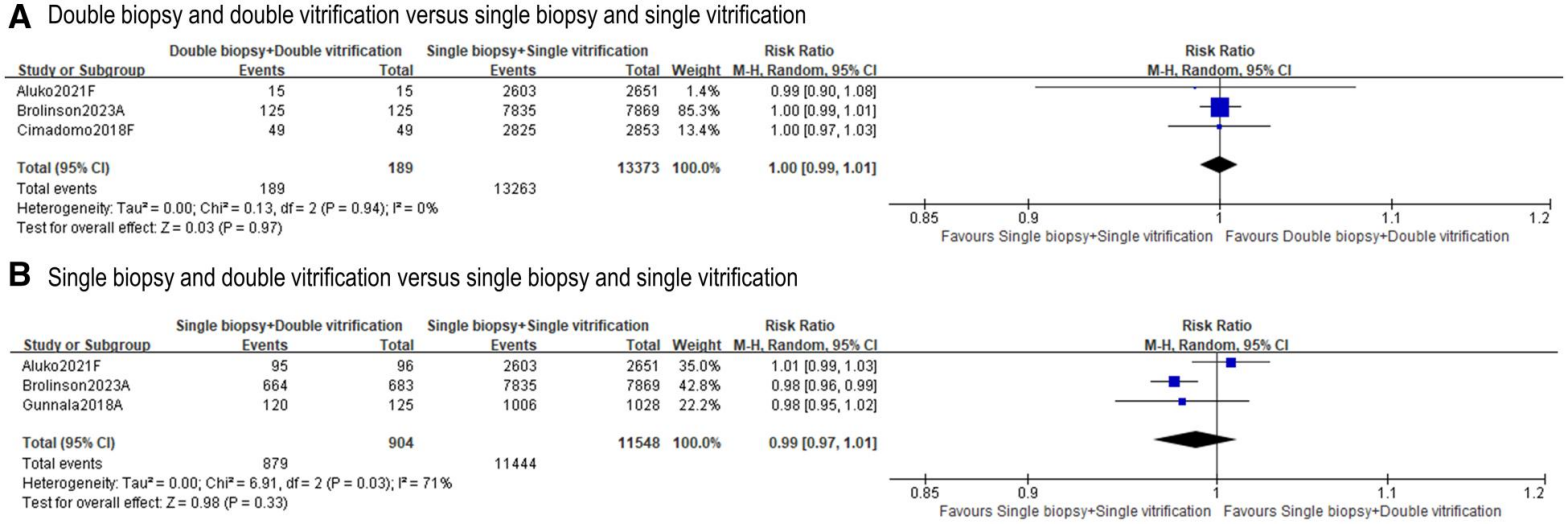
**Effects of ‘double biopsy+double vitrification’ and ‘single biopsy+double vitrification’ on post-warming survival rates**. **A.** Post-warming survival rate following ‘double biopsy and double vitrification’ versus ‘single biopsy and single vitrification’; **B.** Post-warming survival rate following ‘single biopsy and double vitrification’ versus ‘single biopsy and single vitrification’. Study ID: A presents for abstract only, F presents for full-text available. ‘Total’ represents the number of cycles.

Four studies (Cimadomo *et al.*, 2018; Gunnala *et al.*, 2018; Aluko *et al.*, 2021; Brolinson *et al.*, 2023) reported post-warming survival rates. No differences were found when comparing ‘double biopsy + double vitrification’ to ‘single biopsy + single vitrification’ (three studies, n = 13 562; RR 1.00, 95% CI = 0.99–1.01; *I*^2^=0%; Fig. 5A) (Cimadomo *et al.*, 2018; Aluko *et al.*, 2021; Brolinson *et al.*, 2023). Similar results were also observed in ‘single biopsy + double vitrification’ in reference to ‘single biopsy + single vitrification’ (three studies, n = 12 452; RR 0.99, 95% CI = 0.97–1.01; *I*^2^ = 71%; Fig. 5B) (Gunnala *et al.*, 2018; Aluko *et al.*, 2021; Brolinson *et al.*, 2023).

#### Neonatal outcomes

Only one study reported gestational age (weeks) and birthweight (g) (Cimadomo *et al.*, 2018). No differences were found when comparing ‘double biopsy + double vitrification’ to ‘single biopsy + single vitrification’ in gestational age (MD 0.50, 95% CI = −0.09 to 1.09) or birthweight (MD 166, 95% CI = −43.76 to 375.76) (Table 2).

**Table 2.** Results on outcomes not included in meta-analyses.

Outcome	Comparisons (biopsy+vitrification)	Study ID	Study group		Control group		Effect size	95% CI	*P*-value
			Mean/SD	Total	Mean/SD	Total	MD		
Gestational age (weeks)	Double+Double versus Single+Single	Cimadomo2018F	38.8/1.3	19	38.3/1.7	1211	0.50	−0.09 to 1.09	0.10
Birthweight (g)	Double+Double versus Single+Single	Cimadomo2018F	3429/462.7	19	3263/473.9	1211	166	−43.76 to 375.76	0.12

StudyID, A presents for abstract only, F presents for full-text available; MD, mean differences; RR, risk ratio. MD and RR were calculated using RevMan 5.4.

## Discussion

In this systematic review and meta-analysis, we demonstrated the significant impacts of both ‘double biopsy + double vitrification’ and ‘single biopsy + double vitrification’ on the subsequent clinical outcomes following the transfer of euploid blastocysts. The safety of double vitrification in the non-biopsied blastocysts has long been a subject of debate (Stanger *et al.*, 2012; Wang *et al.*, 2023), while in the ‘single biopsy + double vitrification’ scenario, the loss of trophectoderm cells as part of the biopsy process may add further complexity into the understanding of its implications (Aluko *et al.*, 2021). It was proposed by Aluko *et al.* (2021) that, the biopsy timing (i.e. biopsy post-vitrification rather than biopsy fresh) may be a separate factor adversely impacting blastocysts over double vitrification. This is in line with findings in our study, where reduced pregnancy rates and increased miscarriage rates were evident following ‘single biopsy + double vitrification’. In addition, six out of the nine included studies also reported inconclusive results following the original PGT-A, ranging from 2.3% to 4.7% (Table 1). The safety of re-biopsying these blastocysts with an inconclusive result for a re-test remains debatable, considering the additional manipulation and loss of extra trophectoderm cells via a second round of vitrification/warming and biopsy. The quantifiable decreases in pregnancy and live birth rates following ‘double biopsy + double vitrification’ as identified in our meta-analysis offer valuable information in guiding clinical practice. This is particularly useful when counselling patients regarding potential options to handle their inconclusive blastocysts and associated risks.

All nine included retrospective cohort studies investigated ‘double biopsy + double vitrification’ and/or ‘single biopsy + double vitrification’, using ‘single biopsy + single vitrification’ as control. Studies without such a control group were excluded (Aharon *et al.*, 2017; Sekhon *et al.*, 2018). Studies where blastocysts were transferred fresh on Day 6 following biopsy on Day 5 were also excluded from comparison (Colasante *et al.*, 2014). Although some comparisons in our meta-analysis did not reach statistical significance through multivariable regression analyses adjusting for potential confounders, adverse impacts were evident in clinical pregnancy and live birth/ongoing pregnancy rate following ‘double biopsy + double vitrification’ and ‘single biopsy + double vitrification’ (Figs 2 and 3). An elevated miscarriage rate was also observed following ‘single biopsy + double vitrification’ but not ‘double biopsy + double vitrification’ (Fig. 4). This could be partially attributed to smaller sample size in the ‘double biopsy + double vitrification’ analysis (n = 407, Fig. 4A) compared with the ‘single biopsy + double vitrification’ (n = 1243, Fig. 4B). It is also important to highlight that, the observed discrepancy in miscarriage rates between the two study groups may have also been contributed by biopsy operator-related variations and different patient characteristics, which are not addressed in meta-analyses due to the (un)availability of the data. However, post-warming survival seemed to be unaffected by either approach though (Fig. 5). Also, no difference was reported in neonatal outcomes following ‘double biopsy + double vitrification’, which is in agreement with several other reports that were excluded from our meta-analysis due to not meeting inclusion criteria (Bradley *et al.*, 2017; Neal *et al.*, 2018; Li *et al.*, 2023). More definitive conclusions may be drawn from future better-powered statistical analysis as extra data become available.

Significant heterogeneity was observed in our meta-analysis for multiple studied outcomes (Figs 3B, 4A, and 5B). A range of potential factors contributing to the heterogeneity were identified. For example, the Vanderhoff *et al.* (2024) study had stricter criteria for biopsy where only a Gardner score of at least 3BB would be suitable, while a score as low as 3CC (Also Gardner criteria) would be considered by Wilding *et al.* (2019) for biopsy. It is also important to acknowledge the confounding effect of blastocyst grading, post-warming grading, as well as biopsy criteria at both the operator and the clinic levels. While embryo survival is widely defined as >50% of the cells with intact membranes, the embryo assessment criteria represent a significant limitation. In contrast with most other included studies, the Vanderhoff *et al.* (2024) study had the majority of euploid blastocysts transferred on Day 5 with assisted hatching on Day 3. It would therefore be ideal to perform a subgroup analysis based on the day of assisted hatching. However, it is impossible to do so as only one other study (Nohales *et al.*, 2023) from the nine included reported Day 3 assisted hatching, while only Cimadomo *et al.* (2018) reported assisted hatching immediately before biopsy (Table 1). Another potential source of heterogeneity is the age of patients from each study’s population cohort, with a mean age ranging from 35.5 to 37.0 years at oocyte collection in the analysis for ‘double biopsy + double vitrification’ and a mean age from 32.0 to 35.6 years in the ‘single biopsy + double vitrification’ analysis (Table 1). Neal *et al.* (2019) further argued that blastocysts subjected to re-biopsy due to inconclusive PGT-A results were associated with older maternal age, hence the inherently reduced developmental competence. It is also worth noting that not all reported cycles were single blastocyst transfers (Table 1). Last but not least, protocols for both vitrification (including media and carrier device) and biopsy (including number of trophectoderm cells biopsied, use of pulling or flicking technique, whether or not overnight culture post-warm was allowed before biopsy, etc.), skill level of biopsy operator and embryo culture conditions, are highly likely to vary between laboratories involved (Vitale *et al.*, 1997; Cimadomo *et al.*, 2018; Nohales *et al.*, 2023). Indeed, the current standardization of vitrification/biopsy protocol is suboptimal in general, and collective efforts from the entire industry are required to improve the standard. It is therefore paramount for laboratories to keep inconclusive diagnosis under control by monitoring operator competency on a regular basis, ideally monthly, or at least every 100 biopsies. Although the preferred endpoint for such competency assessment should be live birth rate, clinical pregnancy rate may be considered as a surrogate outcome to enable more timely action when needed. In addition, the prevalence of inconclusive diagnosis should also be included as a laboratory PGT key performance indicator, supported by root cause analysis when needed for continuous improvement.

The manipulations and stresses placed on a blastocyst undergoing vitrification and/or biopsy are of significant concern in regard to the subsequent survival and clinical outcomes (Aluko *et al.*, 2021). The same authors further pointed out that the originally tested inconclusive blastocyst could well result in an additional inconclusive result following a re-biopsy due to scarce cellularity or apoptotic cells. There are additional concerns that the embryo may be less likely to be euploid (Neal *et al.*, 2019), despite conflicting views proposed more recently (Córcoles *et al.*, 2022; Vanderhoff *et al.*, 2024). The likelihood of inconclusive blastocysts to be suitable for re-biopsy following warming has been reported to range from 68.9% to 77.4% (Zhang *et al.*, 2014; Parriego *et al.*, 2019; Nohales *et al.*, 2023), where blastocysts deemed not suitable for re-biopsy are mostly discarded. The implantation potential of such ‘drop-out’ blastocyst remains an area to be explored due to the lack of published data. It is also important to highlight that additional labour and expenses are required for a re-biopsy, which are applicable to both the genetics and embryology laboratories. Social economics studies evaluating the cost-effectiveness of this approach are currently lacking in the literature, and certainly warrant future research to provide guiding advice to IVF clinics, especially in countries with PGT funding available.

The strength of our meta-analysis is the exclusion of studies involving a mixture of PGT-A and PGT-SR/M tested blastocysts, minimizing potential confounding impacts by additional patient-related variables. However, due to the retrospective nature of all included studies, selection bias is difficult to avoid. In particular, the ‘double biopsy + double vitrification’ group was more likely to be associated with the poor-prognosis patient population as they were more likely to have experienced failure(s) to achieve pregnancy via single-biopsied euploid blastocysts prior to consideration of the use of re-biopsied euploid blastocysts. Taking this into consideration, an ideal control group to assess clinical outcomes of the ‘double biopsy + double vitrification’ group might be those inconclusive blastocysts following ’single biopsy + single vitrification’, although such data are scarce in existing studies available. Moreover, not all included studies had performed multivariable regression to control potential confounding factors, therefore, only unadjusted data were used in our main meta-analysis. However, available multivariable regression results from included studies were summarized in Supplementary Fig. S2 with adjusted factors listed, and meta-analysis with adjustment was performed where applicable. They were overall consistent with the unadjusted findings. Another limitation is the difficulty to account for variations in biopsy/vitrification protocols and operator skill levels among included studies. A potential publication bias is evident in the funnel plot (Supplementary Fig. S1). Given that it is ethically impractical to test either ‘double biopsy + double vitrification’ or ’single biopsy + double vitrification’ in a clinical setting via randomized controlled trial, the answer to these research questions would still rely on high-quality observational studies. Future large multi-centre studies with adequate approaches to address selection and attrition bias would be important to guarantee better quality data.

## Conclusion

Our meta-analysis, based on a PGT-A dataset, indicates that both ‘double biopsy + double vitrification’ and ‘single biopsy + double vitrification’ were associated with reduced clinical pregnancy and live birth/ongoing pregnancy rates. However, caution ought to be taken when interpreting pooled comparisons due to the inherited limitation in the small sample size of both study groups and the challenge in accounting for all potential confounding factors, such as different patient profiles. To conclude, the biopsy process should be carried out as carefully and competently as possible to minimize the instance of ‘no result/diagnosis’ as a second round of biopsy and vitrification may have an impact on the subsequent clinical outcomes. Patients should also be counselled appropriately on the risks involved before such procedures are conducted.

## Supplementary Material

deae235_Supplementary_Data_File_S1

deae235_Supplementary_Figure_S1

deae235_Supplementary_Figure_S2

deae235_Supplementary_Table_S1

## Data Availability

Data would be made available upon reasonable responses to the corresponding author.
