# Supplementary material for: Impacts of double biopsy and double vitrification on the clinical outcomes following euploid blastocyst transfer: a systematic review and meta-analysis
Source: Hum Reprod. 2024 Oct 7;39(12):2674–84. doi: 10.1093/humrep/deae235 (PMC11630046; doi:10.1093/humrep/deae235)
Supplement: deae235_Supplementary_Data_File_S1 [file deae235_supplementary_data_file_s1.pdf]

OXFORD human reproduction

Human Reproduction, 2024, 1–2
https://doi.org/10.1093/humrep/deae235
Supplementary Data

Supplementary Data File S1. Search strategies and reasons for record exclusion

Search strategies

All databases were searched on 30 August 2023

A. PubMed

1 ‘trophectoderm biopsy’[Title/Abstract] OR ‘re-biopsy’[Title/Abstract] OR ‘rebiopsy’[Title/Abstract] OR ‘second biopsy’[Title/Abstract] OR ‘double biopsy’[Title/Abstract] OR ‘repeat biopsy’[Title/Abstract]
2 Vitrifi*[Title/Abstract] OR cryopreserv*[Title/Abstract] OR Freez*[Title/Abstract]
3 1 AND 2

B. Embase

1 ‘trophectoderm biopsy’:ti, ab OR ‘re-biopsy’:ti, ab OR ‘rebiopsy’:ti, ab OR ‘second biopsy’:ti, ab OR ‘double biopsy’:ti, ab OR ‘repeat biopsy’:ti, ab
2 vitrifi*:ti, ab OR cryopreserv*:ti, ab OR freez*:ti, ab
3 1 AND 2

C. Cochrane Library

1 (‘trophectoderm biopsy’):ti, ab, kw OR (‘re-biopsy’):ti, ab, kw OR (‘rebiopsy’):ti, ab, kw OR (‘second biopsy’):ti, ab, kw OR (‘double biopsy’):ti, ab, kw OR (‘repeat biopsy’):ti, ab, kw
2 (Vitrifi*):ti, ab, kw OR (cryopreserv*):ti, ab, kw OR (Freez*):ti, ab, kw
3 1 AND 2

Reasons for record exclusion when full-text screening and relevant references [n = 28]

A. Not target participants

(not or unclear participants with PGT-A) **[n = 6]**

1. **Carles M**, Sonigo C, Binois O, *et al. Second biopsy for embryos with inconclusive results after preimplantation genetic testing: Impact on pregnancy outcomes*. J Gynecol Obstet Hum Reprod. 2022; 51(8):102436. doi : 10.1016/j.jogoh.2022.102436
2. **De Vos A**, Van Landuyt L, De Rycke M, *et al. Multiple vitrification-warming and biopsy procedures on human embryos: clinical outcome and neonatal follow-up of children*. Hum Reprod. 2020; 35(11):2488–2496. doi : 10.1093/humrep/deaa236
3. **Bradley CK**, Livingstone M, Traversa MV, McArthur SJ. *Impact of multiple blastocyst biopsy and vitrification-warming procedures on pregnancy outcomes*. Fertil Steril. 2017; 108(6):999–1006. doi : 10.1016/j.fertnstert.2017.09.013
4. **Taylor TH**, Patrick JL, Gitlin SA, Michael Wilson J, Crain JL, Griffin DK. *Outcomes of blastocysts biopsied and vitrified once versus those cryopreserved twice for euploid blastocyst transfer*. Reprod Biomed Online. 2014; 29(1):59–64. doi : 10.1016/j.rbmo.2014.03.001
5. *Impact of twice blastocyst biopsies on clinical outcomes*. Leangkonkit, **Duangsamon *et al***. Reproductive BioMedicine Online, Volume 37, e17 (Abstract only)
6. *Rebiopsy and preimplantation genetic screening (PGS) reanalysis demonstrate the majority of originally ‘no diagnosis’ embryos are euploid with comparable pregnancy rates*. **Kaing, A. *et al***. Fertility and Sterility, Volume 104, Issue 3, e277 (Abstract only)

B. Not target comparisons

(no control group, no information on biopsy or vitrification, double biopsy±double vitrification versus single biopsy±double vitrification) **[n = 10]**

1. **D. Johnson**, N. Ramos, Z. Haimowitz, M. Surrey, H. Danzer, J. Barritt, *Embyros with no initial PGT-A result can undergo warming/rebiopsy/revitrification for an attempted reanalysis, however they ultimately demonstrate very low clinical potential*, Reproductive BioMedicine Online, Volume 39, Supplement 2, 2019, Page e10, ISSN 1472–6483, https://doi.org/10.1016/j.rbmo.2019.07.021. (Abstract only)
2. *Inconclusive PGS results: Go for a second biopsy!*. **Parriego, M. *et al***. Human Reproduction, Volume 32, Issue 0, pp. i421 (Abstract only)
3. *Repeat biopsy for preimplantation genetic screening (PGS) reanalysis does not adversely impact obstetrical outcomes*. **Neal, Shelby A. *et al.*** Fertility and Sterility, Volume 109, Issue 3, e41 (Abstract only)
4. *Clinical success with two rounds of vitrification and comprehensive chromosome screening*. **Schlenker, T. *et al***. Fertility and Sterility, Volume 96, Issue 3, S73 (Abstract only)
5. *Analysis of clinical outcome performing fresh or vitrified-warmed blastocyst transfer after trophectoderm biopsy in 307 Preimplantation Genetic Screening with array comparative genomic hybridization cycles*. **Colasante, A. *et al***. Human Reproduction, Volume 29, Issue 0, pp. i89 (Abstract only)
6. *Factors associated with vitrification-warming survival in 6167 euploid blastocysts*. **Oliva, M. *et al***. Journal of Assisted Reproduction and Genetics, Volume 0, Issue 0, pp (Abstract only)
7. *Comparison between fresh or vitrified-warm edblastocyst transfer performed after trophectoderm biopsy in preimplantation genetic diagnosis cycles*. **Ruberti, A. *et al***. Human Reproduction, Volume 28, Issue 0, pp. i303 (Abstract only)
8. *Outcomes following transfer of embryos whose PGT-A results were indeterminate and that were not re-biopsied*. **Alkon-Meadows, T. *et al***. Fertility and Sterility, Volume 114, Issue 3, pp. e429–e430 (Abstract only)
9. *Is reproductive potential compromised when embryos are re-biopsied?*. **Aharon, D. *et al***. Fertility and Sterility, Volume 107, Issue 3, pp. e16–e17 (Abstract only)
10. *Evaluating ivf and perinatal outcomes following repeat trophectoderm biopsy*. **Sekhon, L. *et al***. Fertility and Sterility, Volume 110, Issue 4, pp. e77–e78 (Abstract only)

C. Not target outcomes or data unavailable

(not pre-defined outcomes or data could not be extrated) **[n = 7]**

1. *Repeat biopsy of cryopreserved embryos for preimplantation genetic screening (PGS) reanalysis does not adversely impact reproductive potential*. **Neal, S.A. *et al***. Fertility and Sterility, Volume 108, Issue 3, e276–e277 (Abstract only)
2. *Clinical pregnancy and implantation rates of warmed, biopsied and re-vitrified blastocysts (W-CCS)*. **Popwell, J. M. *et al***. Fertility and Sterility, Volume 101, Issue 2, pp. e20 (Abstract only)
3. *Euploid blastocysts undergoing single vitrification have higher live birth rates than those vitrified twice*. **Bishop, L. A. *et al***. Fertility and Sterility, Volume 110, Issue 4, pp. e421 (Abstract only)
4. *Factors associated with pregnancy loss after single euploid embryo transfer*. **Canon, C. M. *et al***. Fertility and Sterility, Volume 118, Issue 4, pp. e176–e177 (Abstract only)
5. *Neonatal outcomes are not impacted by a second trophectoderm biopsy*. **Kim, J. G. *et al***. Fertility and Sterility, Volume 116, Issue 3, pp. e288 (Abstract only)
6. *Repeated cryopreservation process impairs embryo implantation potential but does not affect neonatal outcomes*. **Wang, M. *et al***. Human Reproduction, Volume 36, Issue 0, pp. i491 (Abstract only)
7. *Double warming and double vitrification for euploid embryos does not affect implantation nor ongoing pregnancy rate*. **Hashimi, B. A. *et al***. Human Reproduction, Volume 36, Issue 0, pp. i231 (Abstract only)

D. Not target objective [n = 1]

1. *Clinical factors associated with monozygotic twinning after single embryo transfer*.**Oliva, M. *et al***. Fertility and Sterility, Volume 114, Issue 3, pp. e281–e282 (Abstract only)

E. Duplicates [n = 4]

1. *Clinical factors associated with thaw survival in a cohort of 6167 vitrified-warmed, euploid blastocysts*.**Oliva, M. *et al***. Fertility and Sterility, Volume 112, Issue 3, pp. e12 (Abstract only, company with B-6 in this list)
2. *Technical and clinical outcomes after 400 rebiopsied blastocysts*. **Córcoles, M. N**. ***et al.*** Reproductive BioMedicine Online, Volume 45, Issue 0, pp. e43–e44 (Abstract only, duplicate with Nohales2023, which in included studies)
3. *What 400 rebiopsied embryos show us: technical and clinical outcomes*.**Córcoles, M. N. *et al***. Fertility and Sterility, Volume 118, Issue 4, pp. e157 (Abstract only, duplicate with Nohales2023, which in included studies)
4. *Inconclusive chromosomal assessment after blastocyst biopsy: Prevalence, causative factors, and outcomes after re-biopsy and re-vitrification Amulticenter experience*.**Cimadomo, D. *et al***. Human Reproduction, Volume 33, Issue 10, pp. 1839–1846 (Abstract only, duplicate with Cimadomo2018, which in included studies)
