# Supplementary material for: Impacts of double biopsy and double vitrification on the clinical outcomes following euploid blastocyst transfer: a systematic review and meta-analysis
Source: Hum Reprod. 2024 Oct 7;39(12):2674–84. doi: 10.1093/humrep/deae235 (PMC11630046; doi:10.1093/humrep/deae235)
Supplement: deae235_Supplementary_Figure_S1 [file deae235_supplementary_figure_s1.pdf]

Human Reproduction, 2024, 1–1
https://doi.org/10.1093/humrep/deae235
Supplementary Data

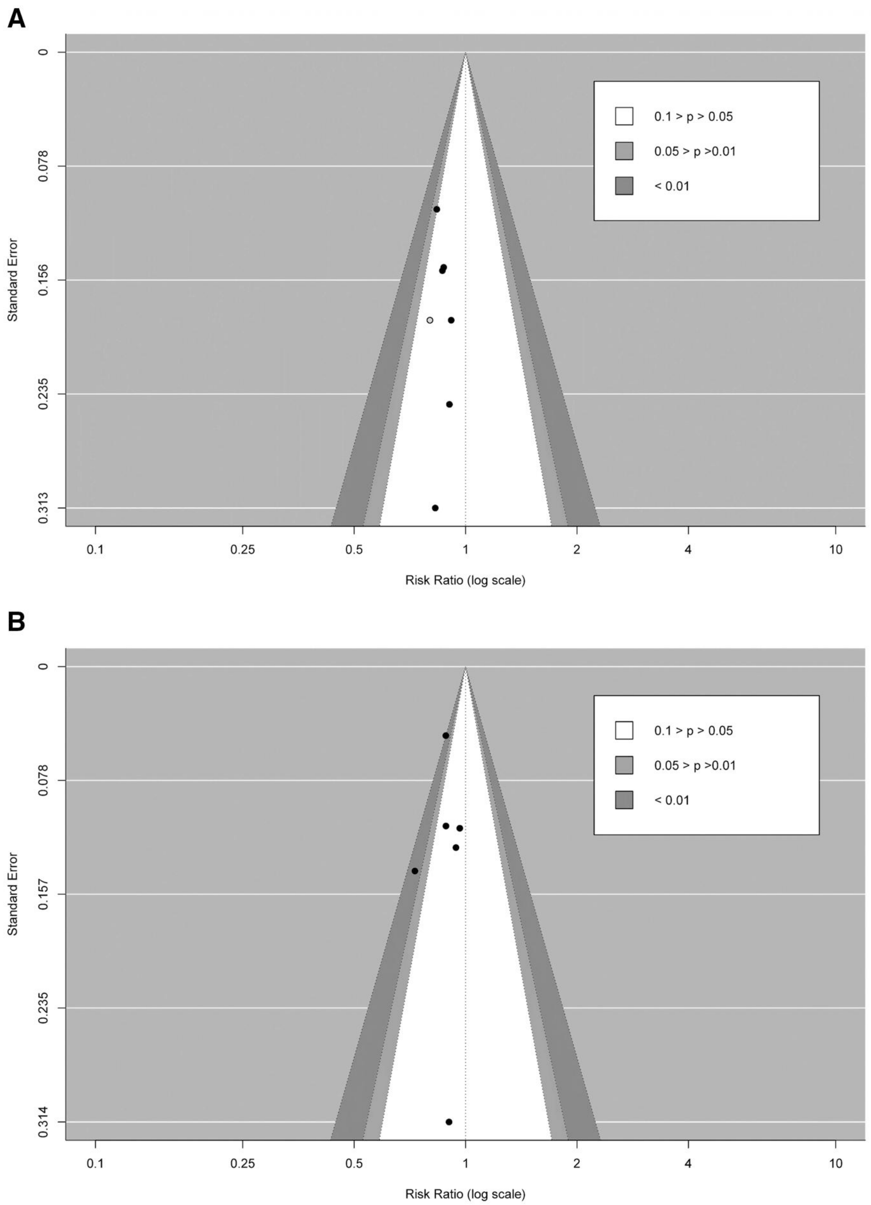

Supplementary Figure S1. Funnel plots for clinical pregnancy rates. (**A**) Double biopsy and double vitrification versus single biopsy and single vitrification. (**B**) Single biopsy and double vitrification versus single biopsy and single vitrification.
