# Supplementary material for: Impacts of double biopsy and double vitrification on the clinical outcomes following euploid blastocyst transfer: a systematic review and meta-analysis
Source: Hum Reprod. 2024 Oct 7;39(12):2674–84. doi: 10.1093/humrep/deae235 (PMC11630046; doi:10.1093/humrep/deae235)
Supplement: deae235_Supplementary_Figure_S2 [file deae235_supplementary_figure_s2.pdf]

Human Reproduction, 2024, 1–1
https://doi.org/10.1093/humrep/deae235
Supplementary Data

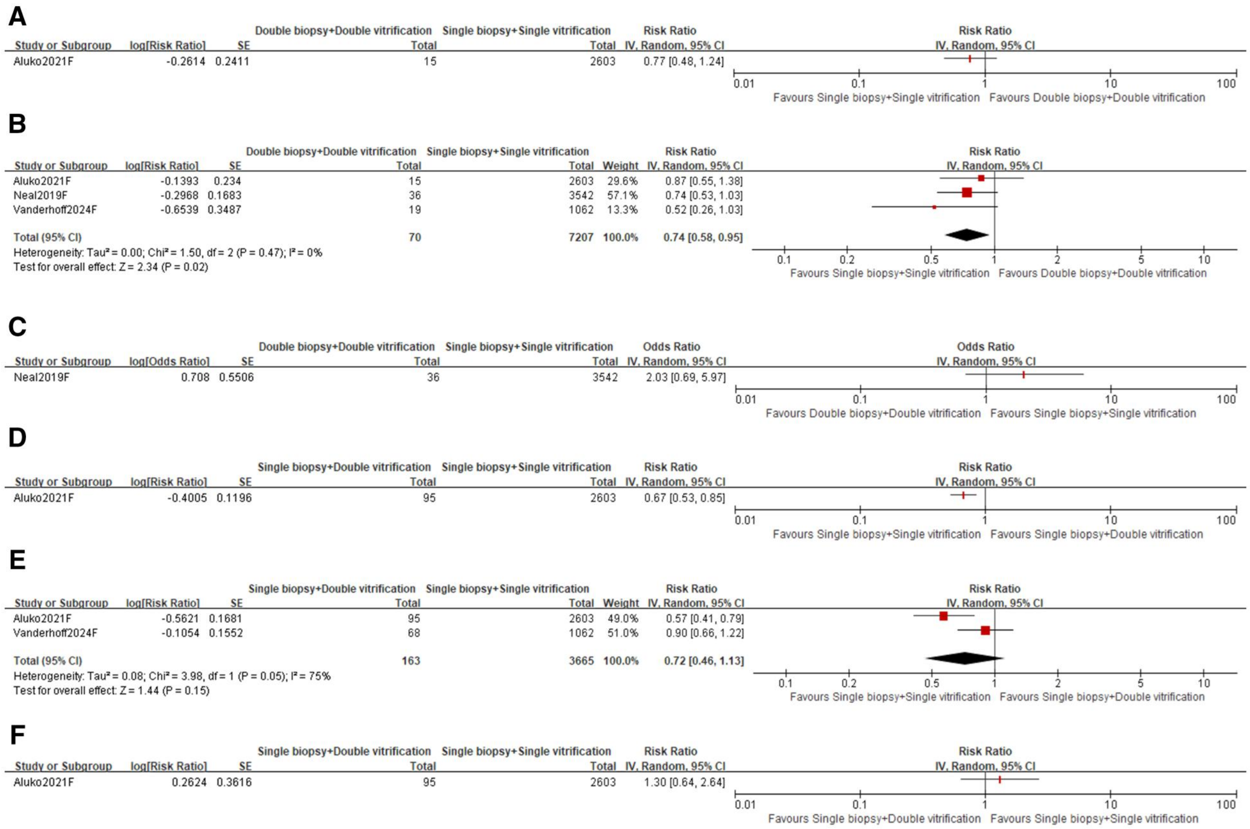

Supplementary Figure S2. Multivariate regression analysis in the effects of 'double biopsy+double vitrification' and 'single biopsy+double vitrification' on different clinical outcomes. (**A**) Double biopsy and double vitrification versus single biopsy and single vitrification for clinical pregnancy rates. Adjusted factors: age at retrieval, number of oocytes retrieved, previous miscarriage, day of embryo vitrification, number of previous cycles, reason for IVF and embryo quality. (**B**) Double biopsy and double vitrification versus single biopsy and single vitrification for live birth and ongoing pregnancy rates. Adjusted factors: Aluko2021F: age at retrieval, number of oocytes retrieved, previous miscarriage, day of embryo vitrification, number of previous cycles, reason for IVF, and embryo quality. Neal2019F: oocyte age and embryo quality. Vanderhoff2024F: oocyte age, BMI, infertility diagnosis, and embryo quality. (**C**) Double biopsy and double vitrification versus single biopsy and single vitrification for miscarriage rates. Adjusted factors: oocyte age and embryo quality. (**D**) Single biopsy and double vitrification versus single biopsy and single vitrification for clinical pregnancy rates. Adjusted factors: age at retrieval, number of oocytes retrieved, previous miscarriage, day of embryo vitrification, number of previous cycles, reason for IVF, and embryo quality. (**E**) Single biopsy and double vitrification versus single biopsy and single vitrification for live birth rates. Adjusted factors: Aluko2021F: age at retrieval, number of oocytes retrieved, previous miscarriage, day of embryo vitrification, number of previous cycles, reason for IVF, and embryo quality. Vanderhoff2024F: oocyte age, BMI, infertility diagnosis, and embryo quality. (**F**) Single biopsy and double vitrification versus single biopsy and single vitrification for miscarriage rates. Adjusted factors: age at retrieval, number of oocytes retrieved, previous miscarriage, day of embryo vitrification, number of previous cycles, reason for IVF, and embryo quality.
