# Supplementary material for: Impacts of double biopsy and double vitrification on the clinical outcomes following euploid blastocyst transfer: a systematic review and meta-analysis
Source: Hum Reprod. 2024 Oct 7;39(12):2674–84. doi: 10.1093/humrep/deae235 (PMC11630046; doi:10.1093/humrep/deae235)
Supplement: deae235_Supplementary_Table_S1 [file deae235_supplementary_table_s1.pdf]

Human Reproduction, 2024, 1–1
https://doi.org/10.1093/humrep/deae235
Supplementary Data

Supplementary Table S1. Risk of bias assessment by the QUIPS tool.

StudyID	Study participation	Study attrition	Prognostic factor measurement	Outcome measurement	Study confounding	Statistical analysis and reporting
Aluko2021F	Moderate risk	Moderate risk	Low risk	Low risk	Low risk[a]	Low risk[a]
Brolinson2023A	Moderate risk	Moderate risk	Moderate risk	Moderate risk	Moderate risk	Moderate risk
Cimadomo2018F	Moderate risk	Moderate risk	Low risk	Low risk	Moderate risk	Moderate risk
Gunnala2018A	Moderate risk	Moderate risk	Moderate risk	Moderate risk	Moderate risk	Moderate risk
Neal2019F	Moderate risk	Moderate risk	Low risk	Low risk	Low risk[a]	Low risk[a]
Nohales2023F	Moderate risk	Moderate risk	Low risk	Low risk	Moderate risk	Moderate risk
Schlenker2019A	Moderate risk	Moderate risk	Moderate risk	Moderate risk	Moderate risk	Moderate risk
Vanderhoff2024F	Moderate risk	Moderate risk	Low risk	Low risk	Low risk[a]	Low risk[a]
Wilding2019F	Moderate risk	Moderate risk	Low risk	Low risk	Low risk[a]	Low risk[a]

QUIPS, Quality In Prognosis Studies; StudyID, First author and publication year, A presents for abstract only, F presents for full-text available.
[a] Studies with abstract only (Brolinson2023A, Gunnala2018A, Schlenker2019A) were assessed as moderate risk in all domains due to limited reported information. These studies addressed key confounders in the design and analysis and were scored as low risk in both domains, but they were scored moderate risk in both domains in unadjusted analysis.

© The Author(s) 2024. Published by Oxford University Press on behalf of European Society of Human Reproduction and Embryology.
This is an Open Access article distributed under the terms of the Creative Commons Attribution License (https://creativecommons.org/licenses/by/4.0/), which permits unrestricted reuse, distribution, and reproduction in any medium, provided the original work is properly cited.
Human Reproduction, 2024, 00, 1–16
https://doi.org/10.1093/humrep/deae235
Meta-Analysis
